# Hypnotism

**Published:** 1891-01

**Authors:** J. R. Callahan

**Affiliations:** Cincinnati, O.


### Hypnotism.

BY J. R. CALLAHAN, D.D.S., CINCINNATI, O.

Read before the Ohio State Dental Society, held at Columbus, October, 1890.

One can scarcely read a daily newspaper now days without seeing article after article devoted to hypnotism, wonderful, and at the same time rediculous, are many of the statements made, all seeming to try to establish the idea that there are persons possessed of some wonderful or divine power over their fellow men. All this confusion is the result of the unfortunate birth of this phenomenon. It fell into the hands of men who were neither capable nor inclined to give it that careful and scientific examination that it deserved.

The terms animal magnetism, mesmerism, clairvoyance, and hypnotism have been used to designate nervous conditions in which the body and mind of an individual were supposed to be influenced by a mysterious force emanating from another person. With the exception of mesmerism, a name given to the phenomena in honor of one of the earliest investigators, F. A. Mesmer, each of those items implies a theory. The phenomena of animal magnetism were supposed to be due to some kind of magnetic force or influence peculiar to living beings and analogous to the action of a magnet upon steel or other metals.

Clairvoyance implied a power of mental vision or of mental hearing or of a mental production of other sensations by which the individual became aware of events happening in another part of the world from where he was, or could tell of the existence of objects which conld not effect at the time any of his bodily senses.

Hypnotism was a name applied to a condition artificially produced, in which a person was apparently asleep, and yet acted in obedience to the will of the operator as regards both motion and sensation.

The power to influence the bodies and minds of others has always attracted much attention and has been sought after for many purposes. We find that whilst not a few have investigated these phenomena in a scientific spirit, more have done so as quacks and charlatans who have thrown discredit on a subject of deepest interest. Recently physiologists and physicians have set about such investigations of the subject as to bring it down into the domain of exact science, and to dispel the idea that the phenomena are due either to any occult force or to supernatural agency. It seems to have been the belief in all ages that the touch, or laying on of hands of certain persons had a healing effect. This power it was supposed for a long time was lodged in priests, and therefore was supernatural and connected with religion.

Frederick Anton Mesmer was born May 23, 1733. He studied medicine at Vienna, took a degree and began practice. Interested in astrology he imagined that the stars exerted an influence on beings living on earth. He identified the supposed force first with electricity, and then with magnetism. He began treating disease by stroking the body with a magnet. In 1766 he observed a priest that produced the same effect without the magnets by manipulation alone. This led Mesmer to discard the magnets and to suppose that some occult force resided in himself. He moved to Paris in 1778, and soon attracted great attention by the effects of mesmerism. Appreciating the effects of mysterious surroundings, he had his consulting apartments dimly lighted and hung with mirrors, strains of soft music occasionally broke the silence, odors were wafted through the room, and the patients sat around a kind of vat in which various chemical ingredients were concocted, or simmered over the fire. Holding each other's hands the patients sat in expectancy. Then Mesmer clothed as a magician glided amongst them, affecting this one by a touch, another by a look, by making passes, etc., etc. He finally fell into discredit and left Paris, and died in 1815.

The use of such methods as Mesmer's were, are used upon the theory that the operator possesses some supernatural power, or that some invisible fluid or influence is given off by the operator that overcomes the patient. The charlatans who travel about the country giving exhibitions usually use some such method, oftentimes adding mysterious gestures and actions to add to the mystery, all of which only proves either ignorance or a desire to deceive.

Dr. H. Bernheim, Professor in the Faculty of Medicine at Nancy, who has given us one of the best works extant on this subject, under the title of Suggestive Therapeutics, says: That the whole explanation lies in suggestion, that is, in the influence exerted by an idea which has been suggested to, and received by the mind. I will give Dr. Bernheim's methods of hypnotizing as briefly as possible. He says: I begin by saying to the patient that I believe benefit is to be derived from the use of hypnotism, that it is possible to cure or relieve him, that there is nothing hurtful or strange about it, that it is an ordinary sleep or torpor which can be induced in every one, and that this quiet, beneficial condition restores the equilibrium of the nervous system, etc. If necessary I hypnotize one or two subjects in his presence in order to show him that there is nothing painful in this condition, and that it is not accompanied by any unusual sensation. When I have thus banished from his mind the idea of magnetism and the somewhat mysterious fear that attaches to that unknown condition, above all, when he has seen patients cured or benefitted by the means in question, he is no longer suspicious, but gives himself up; then I say, look at me and think of nothing but sleep, your eyelids begin to feel heavy, your eyes tired, they are getting moist, you can not see distinctly, they are closed. Some patients close their eyes and are asleep immediately. With others I have to repeat, lay more stress on what I say and even make gestures, it makes little difference what sort of gesture is made. I persuade him to fix his eyes on mine, endeavoring at the same time to concentrate his mind on the idea of sleep. I say, your lids are closing, you can not open them again, your arms feel heavy so do your legs, you can not feel any thing, you see nothing, you are going to sleep, and I add in a commanding tone, sleep. This word often turns the balance. I use the word sleep in order to obtain as far as possible over the patients a suggestive influence which shall bring about sleep, or a state closely approaching it, for sleep, properly so-called, does not always occur. Finally, after the eyes close I keep repeating the suggestion, your lids are stuck together, you can not open them, the need of sleep becomes greater and greater, you can no longer resist. I lower my voice gradually, repeating the command, sleep, and it is very seldom that more than three minutes pass before sleep, or some degree of hypnotic influence is obtained. It is sleep by suggestion, a type of sleep which I insinuate into the brain.

This brief extract from Dr. Brenheim's method does not by any means cover all cases. I give this much of this modern method to show that the mask of hypocracy and pretension has been taken from this phenomena, and that hypnotism is being practiced upon an apparently sound basis, and is no longer one of the curiosities of science.

The condition of the patient under the influence of hypnotism is seemingly normal, in some cases there is an acceleration of the cardiac and respiratory movements, this, however, can be accounted for from the fact that the patient is always more or less excited when they submit to an operation of any kind for the first time. In patients who have been hypnotized a number of times, the action of the heart and lungs remains natural according to the best authorities.

As to who, and what per cent. of people can be brought under this influence, M. Siebault gives us the best statistics. In 1880, he hypnotized 1,012 persons. From his figures it appears that the proportions of subjects that can be hypnotized are about the same in men as in women, viz., 18.8 per cent. in men, 19.4 per cent. in women. A striking point in this table is the great proportion of subjects in children and youths of the ages from 1 to 7 years, 26.5 per cent.; of the ages from 8 to 14 years, 55.3 per cent. were successfully hypnotized. In old age the number decreases, but always remains at the relatively high figure of 7 to 11 per cent.

Of what practical benefit is this phenomena is a question that I can not answer in one short paper even if I possessed the knowledge to do so. It has been used successfully in many cases of organic diseases of the nervous system, hysterical diseases, neuropathic affections, rheumatic affections, etc.

Its application to dentistry has, so far, been very limited, because it has been the aim of dentists to produce an entire insensibility to pain. This has been done only in a small per cent. of cases. It has been almost impossible to bring the patient to the anæsthetic condition while he has the idea of an operation before him. Along with the many failures in this respect we have many very successful surgical operations reported where hypnotism was the only anæsthetic employed. Among others Drs. Reibold and Kiaro, dentists, as far back as 1847, removed a tumor of the jaw, painlessly, while the patient was under hypnotic influence, but as said before, hypnotizing generally fails with persons disturbed by the expectation of an operation.

In my opinion, careful investigation on the part of the dental profession would soon develop methods that would prove highly beneficial, but who is going to undertake such a work in the face of such strong public prejudice as exists to-day in regard to this subject? It amounts to superstition. If a dentist was known to be a professional hypnotist the public would at once put him down as a fanatic, a crank, a spiritualist, an infidel, and all that follows in that line. So a man who cares for his reputation has to keep rather quiet on such a subject. Then, too, there are dangers connected with the unskillful and dishonest use of this phenomena. After having been hypnotized a certain number of times, some subjects preserve a disposition to go to sleep spontaneously at most inconvenient times, others are too easily susceptible to hypnotization when they have been too often subjected to it. Such susceptibility is the real danger. This unpleasant tendency can, and should, be controlled by the skillful and sincere operator by suggestion.

An ordinary office practice is too limited a field, and in other ways an objectionable field in which to carry on the investigation of the subject. The large clinics at our dental colleges would be the proper place for such work.

May I express the hope that soon we may have some reliable information on this point from the source indicated?

